# Experimental and Finite Element Research on the Failure Mechanism of C/C Composite Joint Structures under Out-of-Plane Loading

**DOI:** 10.3390/ma12182922

**Published:** 2019-09-10

**Authors:** Yanfeng Zhang, Zhengong Zhou, Zhiyong Tan

**Affiliations:** 1 National Key Laboratory of Science and Technology on Advanced Composites in Special Environments, Harbin Institute of Technology, Harbin 150080, China; 2 Beijing Institute of Near-space Vehicle’s System Engineering, Beijing 100076, China

**Keywords:** C/C composites, out-of-plane loading, finite element, progressive damage, failure mechanism

## Abstract

The loading and the failure mode of metal hexagon bolt joints and metal counter-sunk bolt joints of C/C composites were investigated. The joints were tested for out-of-plane loading at two temperatures (600 °C and 800 °C). The failure morphology of a lap plate was investigated, and the main failure modes were determined. The typical load–displacement curve was characterized and the test was simulated using ABAQUS non-linear finite element software. Furthermore, progressive damage was induced, and comparison of the finite element simulation with the experimental data revealed that the failures mainly occurred in the lower lap plate and were dominated by cracking and delamination of the matrix, accompanied by the pull-out of a small number of piercing fibers. Finally, the influences of the temperature, nut radius, and fixture geometry on the critical load were determined via simulation.

## 1. Introduction

C/C composites are high performance materials with carbon fiber textiles as the reinforcing element and carbon resin, asphalt carbon, or deposited carbon as the matrix [1]. Due to their low density, low thermal expansion coefficient, and excellent mechanical performance at high temperatures, C/C composites are key thermal structural materials for aerospace applications [2,3,4,5,6] and are already used in the nose cone cap, the wing leading edge, and turbine engine components of airplanes. Since mechanical joints are essential but weak parts of these structures, C/C composites have been widely used in the joint structures of aircrafts operating in harsh environments, such as hypersonic vehicles, to enhance the safety and stability of the joints. 

**Figure 1:**
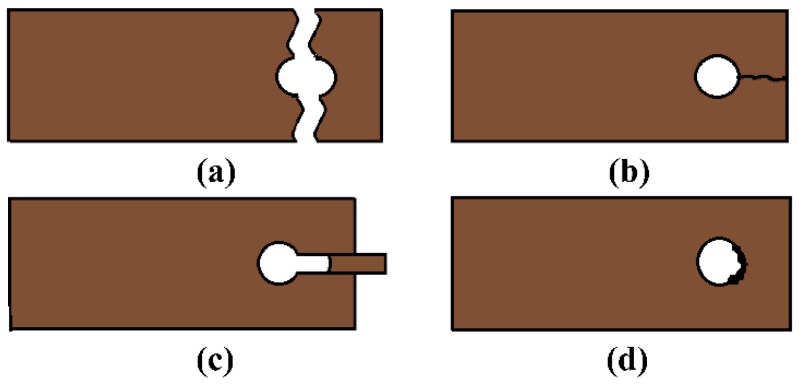
Common in-plane failure modes of bolt joint structures: (**a**) tensile failure, (**b**) splitting failure, (**c**) shear failure, and (**d**) extrusion failure.

**Figure 2:**
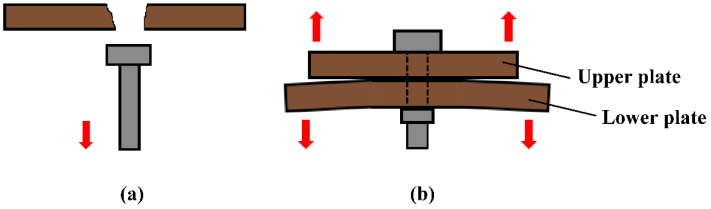
Common out-of-plane failure modes of (**a**) single plate bolt joint structure and (**b**) a double plate bolt joint structure.

To date, various studies on the bearing capacity and failure mode of joint structures of composites have been carried out, and the methods involved include experimental testing [7,8], finite element simulations [9,10,11], and analytical methods [12,13]. The previous work focused on the factors affecting the performance of single bolt joint structures [14,15], multiple bolt joint structures [16,17,18], and composite joint structures. These factors include the geometrical configuration of the fasteners [19], the supporting forces of the specimen [20], and the layering sequence [21]. All previous reports were limited to joint structures of resin-based composites reinforced by fibers with a temperature tolerance below 400 °C. However, structures with a temperature tolerance above 400 °C were rarely discussed. In particular, most existing studies on joint structures with C/C composites focused on their performance at room temperature, and studies at high temperatures are extremely limited [22]. For instance, most studies discussed the performance of the C/C composites at room temperature only [23,24], not the overall performance of the C/C composite structures. This has two origins. First, C/C composite joints are state-of-the-art technologies in military research, and only a few studies are eventually published. Second, the C/C composites are limited by their complicated preparation process [25], high cost, and their easy oxidation at high temperatures [26]. Additionally, most studies on composite joint structure have been related to the failure of the joints under a load parallel to the lap plate (Figure 1) [7,8,9,10,11,12,13], but only a few have discussed the failure of the joints under a load perpendicular to the lap plate (Figure 2). Consequently, the bearing capacity determined in these studies is minimized along the thickness of the lap plate. On the contrary, this study investigates the performance and failure mode of metal bolt joints made from C/C composites under an out-of-plane load at high temperatures using experimental data and finite element simulations. 

## 2. Materials and Methods 

### 2.1. Materials and Configuration

**Figure 3:**
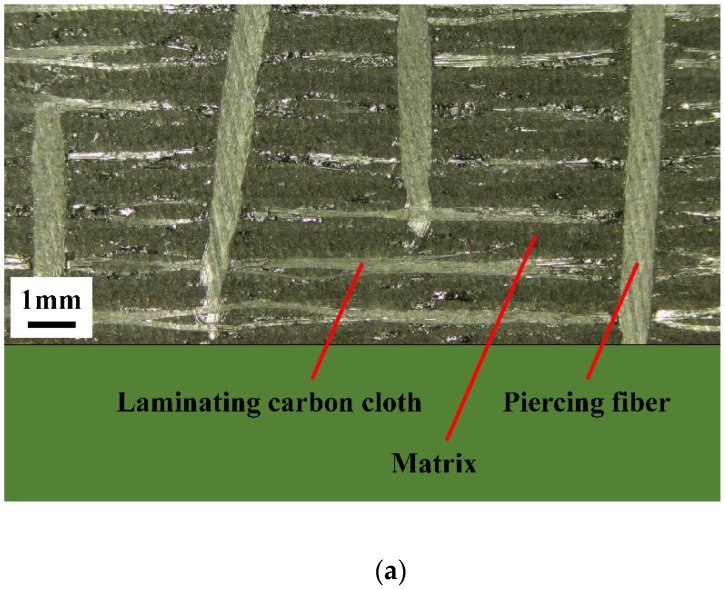

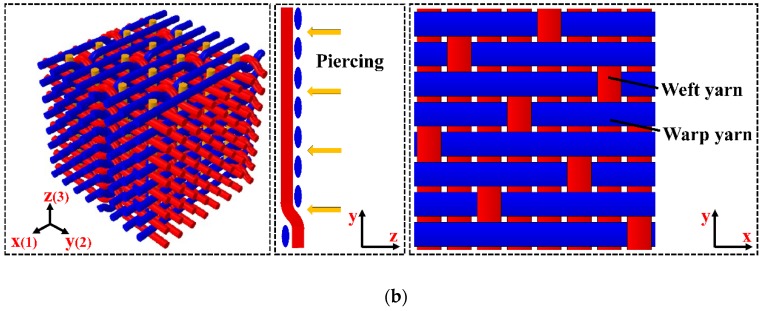
Layer structure of the C/C composites (**a**) and braiding pattern of the pre-fabricated part (**b**).

**Figure 4:**
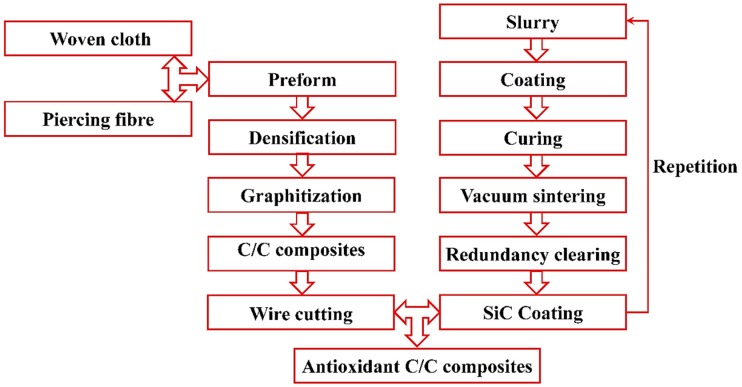
Preparation of the coated C/C composite plates.

**Figure 5:**
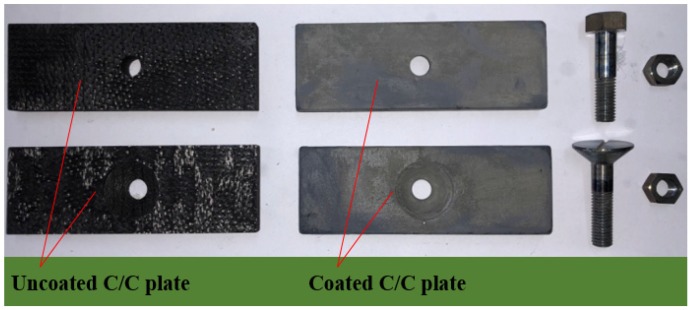
Lap plate, fasteners, and their lap mode.

**Figure 6:**
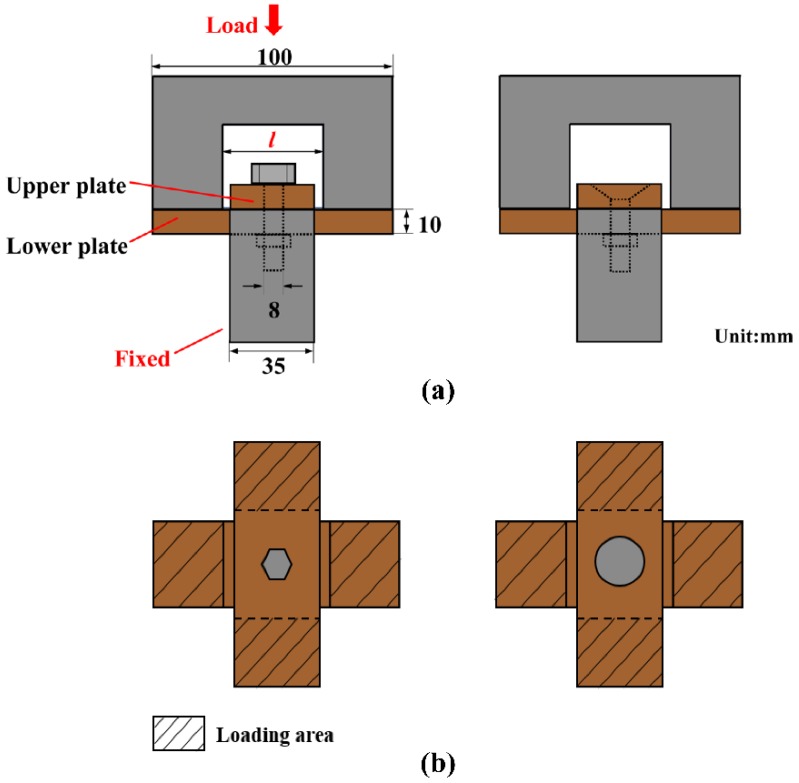
Loading pattern and specimen size.

The lap plates were made of C/C piercing composites. The carbon fiber cloth was placed on the (x, y) plane of the pre-fabricated part by braiding and was stacked along the z direction, The composite was reinforced in the z direction by piercing carbon fibers, as shown in Figure 3a. The stacked carbon fiber cloths and the z direction fibers were eight satins (T300-3K by Toray, Tokyo, Japan) and carbon fibers (T300-1K by Toray, Japan) with a piercing distance of 4.5 mm. Figure 3b shows the braiding pattern and the fiber orientation in the pre-fabricated part. The x, y, and z directions correspond to the three main directions of the material. The pre-fabricated parts were densified by chemical vapor infiltration (CVI, Jiang Tai Vacuum Coating Technology Ltd., Chengdu, China) [27,28] and graphitized to obtain the C/C composites. According to previous studies [29], C/C composites are readily oxidized in air above 400 °C, and the oxidation rate is proportional to the temperature. In order to avoid the destruction of the specimens, the C/C plates were coated with silicon carbide by chemical vapor deposition (CVD, Penta Technology Co., Ltd., Suzhou, China) [30] to obtain lap plates suitable for high-temperature testing. The procedure is summarized in Figure 4. The fasteners were made out of Inconel 600 Nickel superalloy and can be described as hexagon bolts and countersunk bolts. In previous studies on out-of-plane loading failures of composites, the loads were applied directly on the bolts that pierced the composite plate. Consequently, the specimens had a single-plate structure, as shown in Figure 1a. Practically, however, most joints have a double-plate structure (Figure 1b). The bearing capacity of the structure is usually severely reduced before piercing the lap plate with the bolt. In this study, the long sides of two rectangular C/C plates with a centered opening were perpendicular to each other and formed a cross in single lap mode. The specimens can be described as hexagonal alloy bolt joints and countersunk alloy bolt joints. Figure 5 shows the lap plate and the fastener as well as the lap mode before and after the antioxidation treatment. In order to minimize the effects of the mismatch in the thermal properties of the different materials on the experimental results, a pair of symmetric U-shaped superalloy fixtures was used, and the upper and lower plates were in contact with the fixtures. The distance between the forced areas of a plate was *l* (here, *l* = 42 mm). The loads were applied on the two end faces (Figure 6). In this study (<1000 °C), the modulus and the strength of C/C composites in all directions were proportional to the temperature, although the variation was low. Table 1 illustrates the performance parameters of C/C composites at room temperature, at 600 °C, and at 800 °C, as provided by the Aerospace Research Institute of Materials and Processing Technology in Beijing. However, in order to obtain the simulation results at other temperature points, processing was simplified and the relevant material parameters were obtained at other temperature points by the interpolation method. Of course, the relevant results need to be further verified.

### 2.2. Procedures

**Figure 7:**
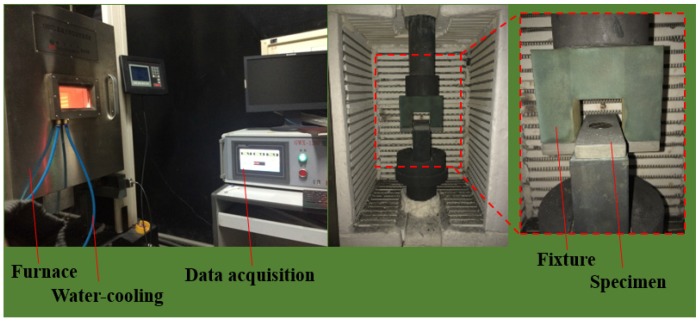
Test equipment and specimen fixture.

The experimental tests were carried out on a WDW-100 computer-controlled electronic universal system (SINGO Technology (Hangzhou) Co., Ltd. Hangzhou, China) equipped with a high-temperature furnace, an automatic data acquisition system, and a water-cooling system (Figure 7). The fixture was in contact with the upper and lower plates to transfer loads. The high-temperature and quasi-static compression failures of the joints were tested.

First, the temperature in the furnace was increased to the required temperature (600 and 800 °C), respectively. The heating rate was 15 °C/min. At 800 °C, the test piece showed slight redness. Then, loads were applied at 0.5 mm/min until the bearing capacity of the specimen was minimized. The loads were controlled by the displacement. Due to the high-cost and the complicated procedure required to obtain the C/C composites, the specimens were only tested at 600 and 800 °C.

## 3. Results and Discussion

### 3.1. Failure Mode 

**Figure 8:**
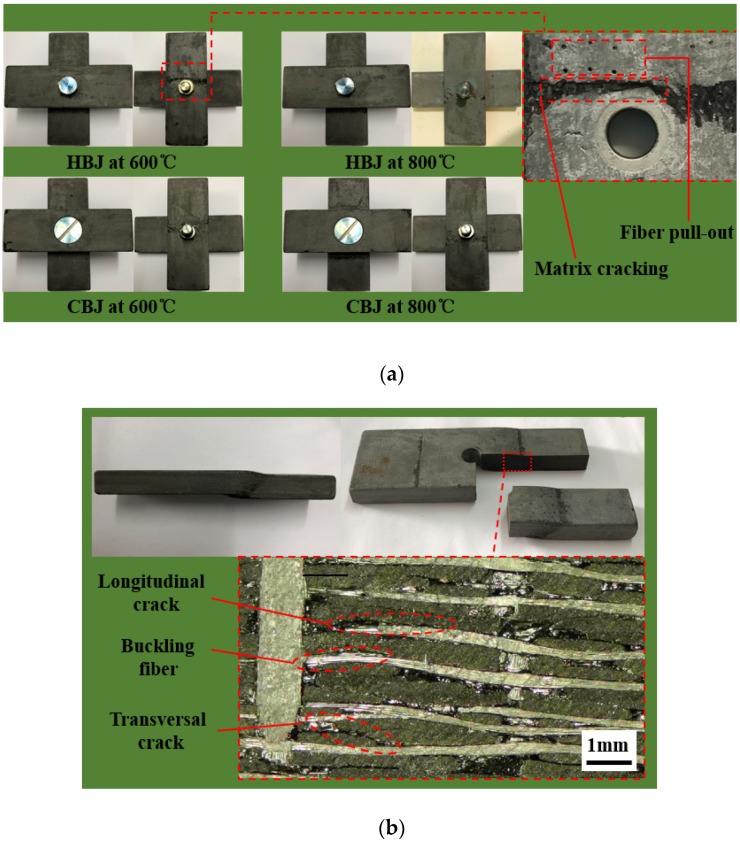
Failure morphology of the (**a**) top and bottom surfaces and the (**b**) side of the lower lap plate.

**Figure 9:**
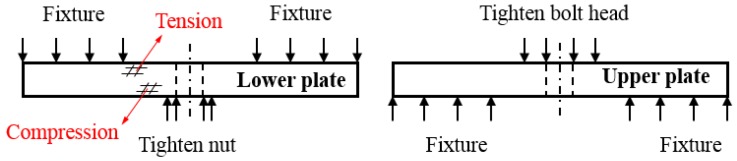
Load on the lap plate.

Figure 8a shows the failure morphologies of the top and bottom surfaces of the hexagonal bolt joints and countersunk bolt joints at two different temperatures. The damaged areas of both joints are indicated on the lower lap plate and are distributed along the width of the rectangular plate. During loading, the upper and lower lap plates were clamped by the bolt head and the nut, respectively, in addition to the fixture pressure (Figure 9). Since the working area of the bolt head was larger than that of the nut, the bending moment on the lower plate was larger than that on the upper plate. Therefore, damage was mainly observed on the lower plate. The damaged area of the lower plate was characterized using a stereomicroscope (Olympus SZ-STU2, Tokyo, Japan). Matrix cracking induced by the compression, the shear, and the pulling out of piercing carbon fibers was observed along the plate thickness. Figure 8b illustrates the failure morphology of the side of the lower lap plate. Bending and dislocations were observed in the lower lap plate, indicating that the joints were mainly exposed to mixed bending/shear failure. Types of damage included cracking and delamination of the matrix. The angle between the damaged area and the plate length was approximately 45°. The delamination was attributed to two factors. First, the crack propagated along the plate thickness near the fixture and the nut when the matrix was under compression. Since the modulus and the strength of the matrix were lower than those of the fibers, the propagation of the cracks moved to the inter-layer interface once they approached the fibers, which created delamination. The first consequence was the buckling of the fibers along the plate length in the compressed area induced by the bending effect. Meanwhile, the deformations of pulled and compressed fibers by bending were different (Figure 9), resulting in a change in the relative locations of the fibers on a specific cross-section and relative dislocations of the single layer materials. The delamination developed in this way. The pulling-out of piercing fibers shown in Figure 9a can also be attributed to the degradation of the binding between the piercing fibers and the matrix induced by the delamination cracks, which may have led to the separation of the piercing fibers and the matrix as well as the carbon fiber cloth. 

### 3.2. Numerical Simulation

C/C composites are high-temperature materials whose performance depends on the temperature. Due to limitations in the number of specimens available, experiments alone cannot reveal the bearing capacity of C/C composite joints. Therefore, finite element numerical simulations of the loading of C/C composite joints were carried out using ABAQUS 6.14 (Dassault, Paris, France). Then, the results were compared with experimental data. This allowed verification of the model, analysis of the joint failure, and investigation of the influence of the temperature on the critical load of the joint. 

**Figure 10:**
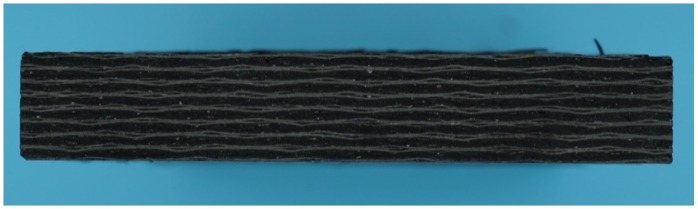
Number of plate layers.

**Figure 11:**
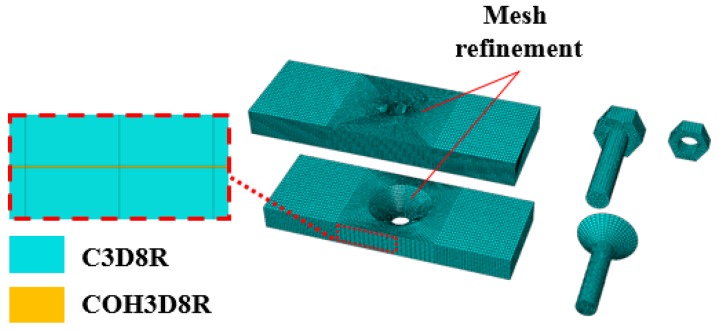
Mesh generation.

**Figure 12:**
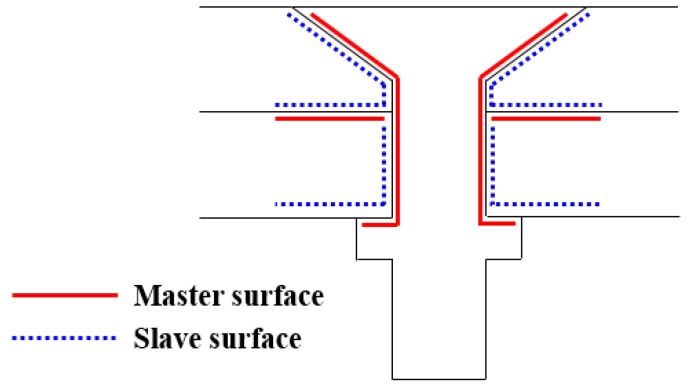
Master and slave surfaces in each contact pair.

The plates in the C/C composite joints were divided into 16 layers along their thickness according to their geometry (the actual number of layers was 16, as shown in Figure 10). Each layer was then individually modeled to show the bending during loading, to represent a C3D8R unit that is not readily exposed to shear self-locking under flexural loading. Therefore, the displacements obtained were accurate and the effect of mesh distortion on the accuracy of the analysis was negligible. A cohesive unit was inserted between each pair of layers to simulate damage due to delamination. Since the accuracy of finite element calculations is significantly affected by the mesh quality, the mesh was refined near the opening. The bolts and the fixtures were considered to be stiff materials, since their deformation during loading was negligible. During loading, temperature loads were applied using a pre-defined field. The initial temperature was the room temperature, and the sample was then heated to the set temperature. Figure 11 shows the mesh generation. The contacts in the model were achieved by defining contacting surfaces and master and slave surfaces for each contact pair, as shown in Figure 12. 

In this study, damage to the C/C composites was determined using the Hashin criteria [31]. Previous studies showed that the failure behavior of opening-containing composites can be predicted by the Hashin criteria [32]:

(1) Tensile failure of the fibers ($\sigma_{11}>0$):

(1) $${\left(\frac{\sigma_{11}}{X_{T}}\right)}^{2}\geq 1;$$

(2) Compressive failure of the fibers ($\sigma_{11}<0$):

(2) $${\left(\frac{\sigma_{11}}{X_{C}}\right)}^{2}\geq 1;$$

(3) Tensile failure of the matrix ($\sigma_{33}>0$):

(3) $${\left(\frac{\sigma_{22}}{Y_{T}}\right)}^{2}+{\left(\frac{\tau_{12}}{S_{12}}\right)}^{2}\geq 1;$$

(4) Compressive failure of the matrix ($\sigma_{22}<0$):

(4) $${\left(\frac{\sigma_{22}}{Y_{C}}\right)}^{2}+{\left(\frac{\tau_{12}}{S_{12}}\right)}^{2}\geq 1$$

where $\sigma_{ij}$(*i*, *j* = 1~2) is the stress tensor, $X_{T}\;\mathrm{and}\;X_{C}$ are the tensile and compressive strengths in the ***x*** direction, $Y_{T}\;\mathrm{and}\;Y_{C}$ are the tensile and compressive strengths in the ***y*** direction, and $S_{12}$ is the in-plane shear strength (Table 1). 

In the presence of damage, the performance parameters of the composites degrade, and this must be taken into account in the analysis of progressive damage. 

To reflect the material degradation, a damage factor (*d*) was introduced:

(5) $$d=\frac{\delta_{eq}^{f}\left(\delta_{eq}-\delta_{eq}^{0}\right)}{\delta_{eq}\left(\delta_{eq}^{f}-\delta_{eq}^{0}\right)}$$

where $\delta_{eq}^{0},\;\delta_{eq},\;\delta_{eq}^{f}$ refer to the initial, current, and final equivalent displacements, respectively. 

If any of the criteria listed earlier are valid, damage occurs and the stiffness degrades. The degraded stiffness matrix is

(6) $$C_{d}=\frac{1}{D}\left[\begin{array}{ccc} \left(1-d_{f}\right)E_{1} & \left(1-d_{f}\right)\left(1-d_{m}\right)\nu_{21}E_{1} & 0\\ \left(1-d_{f}\right)\left(1-d_{m}\right)\nu_{12}E_{2} & \left(1-d_{m}\right)E_{2} & 0\\ 0 & 0 & \left(1-d_{s}\right)G_{12}D \end{array}\right]$$

where *E*_1_, *E*_2_, *G*_12_, *ν*1_2_, *ν*_21_ are the parameters given in Table 1, $d_{f}\;\mathrm{and}\;d_{m}$ are damage variables derived from $d_{f}^{t},\;d_{m}^{t},d_{f}^{c},d_{m}^{c},$ and $d_{s}$ is the shear damage variable expressed as $d_{s}=1-\left(1-d_{f}^{t}\right)\left(1-d_{m}^{t}\right)\left(1-d_{f}^{c}\right)\left(1-d_{m}^{t}\right)\left(1-d_{f}^{t}\right),D=1-\left(\left(1-d_{f}^{}\right)\left(1-d_{m}^{}\right)\;\nu_{12}\nu_{21}\right)$.

The cohesion area model was established based on the stress–displacement constitutive relation of the unit and is commonly used to simulate the linear behavior and the subsequent softening behavior and to calculate the stiffness degradation up to the failure. Indeed, the cohesion area model has been widely used to analyze the failure of composites [33,34]. In this study, a double linear constitutive relation of the cohesion unit was used to simulate delamination failure. 

**Figure 13:**
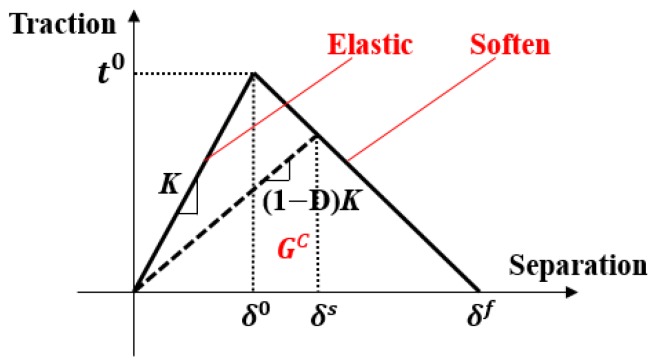
Double linear constitutive relation.

As shown in Figure 13, the double linear constitutive relation assumes that the stress–displacement of the unit remains linear, regardless of the intensity of the stress relative to the material strength. In the absence of damages to the unit, the constitutive relation is

(7) $$\left[\begin{array}{c} t_{n}\\ t_{s}\\ t_{t} \end{array}\right]=diag(K_{nn},\;K_{ss},\;K_{tt})\left[\begin{array}{c} \varepsilon_{n}\\ \varepsilon_{s}\\ \varepsilon_{t} \end{array}\right].$$

The linear elasticity can be expressed by the stress–strain constitutive matrix of the unit:

(8) $$t={\left[\begin{array}{ccc} t_{n} & t_{s} & t_{t} \end{array}\right]}^{-1},\;\varepsilon ={\left[\begin{array}{ccc} \varepsilon_{n} & \varepsilon_{s} & \varepsilon_{t} \end{array}\right]}^{-1}$$

where $t_{n},\;t_{s},\;t_{t}$ are the normal component and the two tangential components of the stress on the unit, respectively. $\varepsilon_{n},\;\varepsilon_{s},\;\varepsilon_{t}$ are the normal component and the two tangential components of the strain of the unit. The stiffness matrix $K={\left[\begin{array}{ccc} K_{nn} & K_{ss} & K_{tt} \end{array}\right]}^{-1}$ contains the stiffness parameters of the inter-layer interface of the composites. When the external stress increases, the opening displacement of the unit increases. Once the displacement reaches the critical damage level ($\delta^{0}$), the stress reaches the strength in this direction ($t^{0}$). Then, damage to the unit occurs and the material softens. ABAQUS provides several criteria for the initial damage, but we used the maximum stress criteria:

(9) $$\max\left\{\frac{\langle t_{n}\rangle }{t_{n}^{0}},\;\frac{t_{s}}{t_{s}^{0}},\;\frac{t_{t}}{t_{t}^{0}}\right\}=1$$

where $t_{n}^{0},\;t_{s}^{0},\;t_{t}^{0}$ are the normal strength and the shear strengths in both directions of the unit.

If the maximum nominal stress ratio in all directions in the cohesion unit is equal to 1, damage occurs. The constitutive relation at the softening stage is

(10) $$\left[\begin{array}{c} t_{n}\\ t_{s}\\ t_{t} \end{array}\right]=\left(1-D\right)diag(K_{nn},\;K_{ss},\;K_{tt})\left[\begin{array}{c} \varepsilon_{n}\\ \varepsilon_{s}\\ \varepsilon_{t} \end{array}\right]$$

where D is the damage variable (0≪D≪1; D=0 indicates no damage and D=1 indicates complete failure). In the case of complete failure, the opening displacement of the unit is equal to the critical displacement ($\delta^{f}$), which is determined by the rate of critical strain energy release in the unit ($G^{C}$). $G^{C}$ is reflected in the area between the curve and the *x* axis. Cohesion element parameters were supplied by the Aerospace Research Institute of Materials and Processing Technology in Beijing. The input parameters of other temperature points required for numerical simulation were also obtained by interpolation method. Of course, the relevant results also need to be further verified. The values of parameters required for calculation are shown in Table 2.

### 3.3. Progressive Damage

To fully understand the out-of-plane loading failure of joints, the experimental load–displacement curves were compared with finite element calculations to investigate the progressive damage to the joints. 

**Figure 14:**
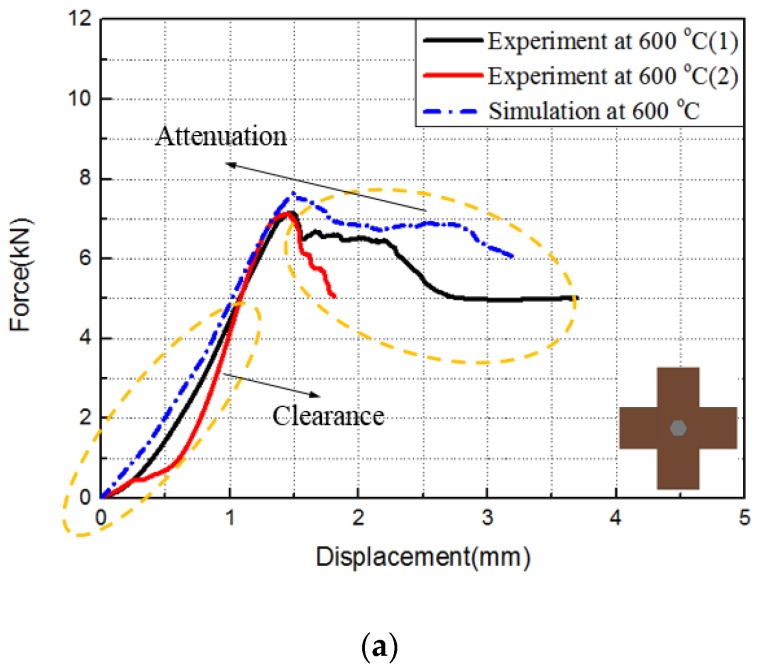

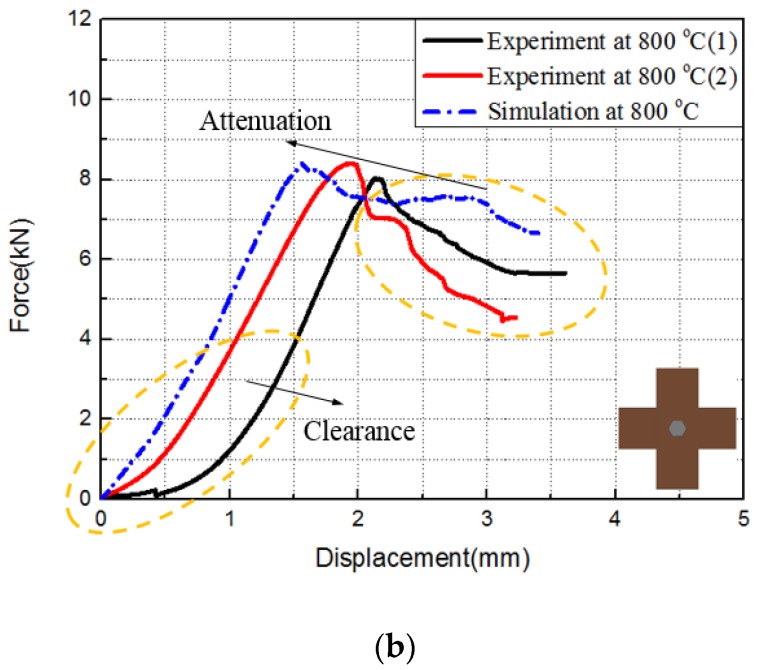
Force–displacement curves of hexagonal bolt joints at (**a**) 600 °C and (**b**) 800 °C.

**Figure 15:**
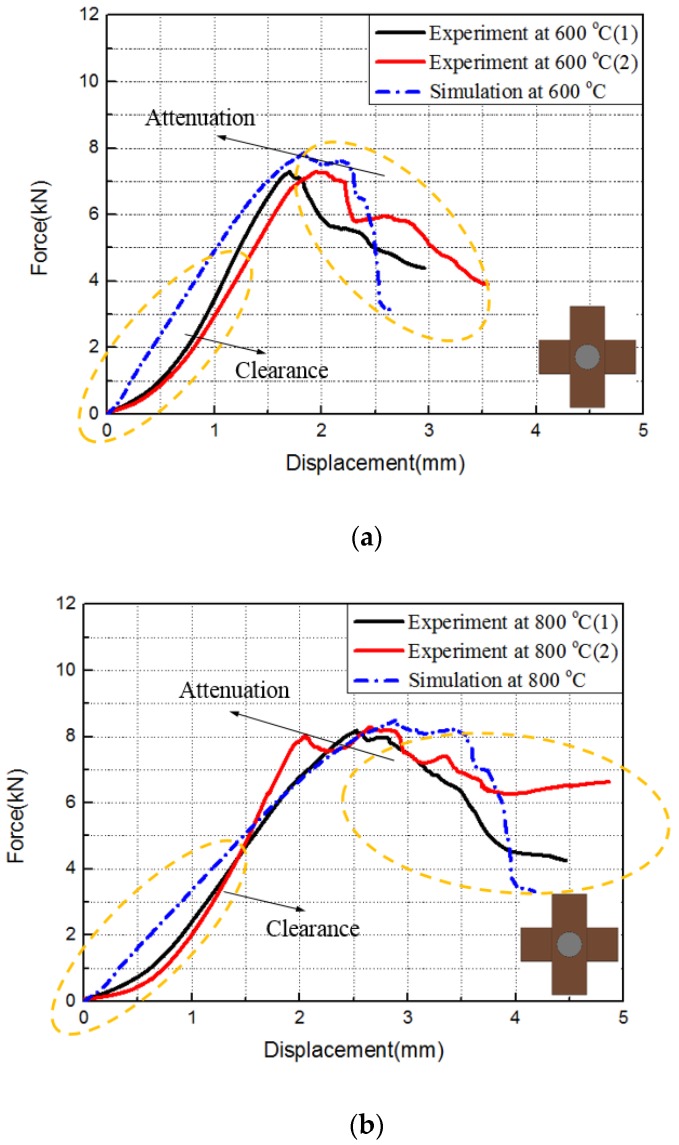
Force–displacement curves of the countersunk bolt joints at (**a**) 600 °C and (**b**) 800 °C.

**Figure 16:**
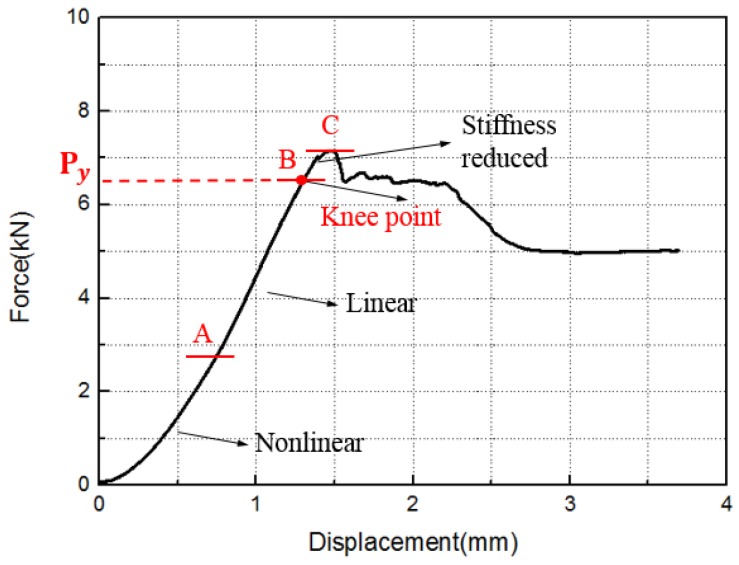
Initial load–displacement curve of the hexagonal bolt joints at 600 °C.

**Figure 17:**
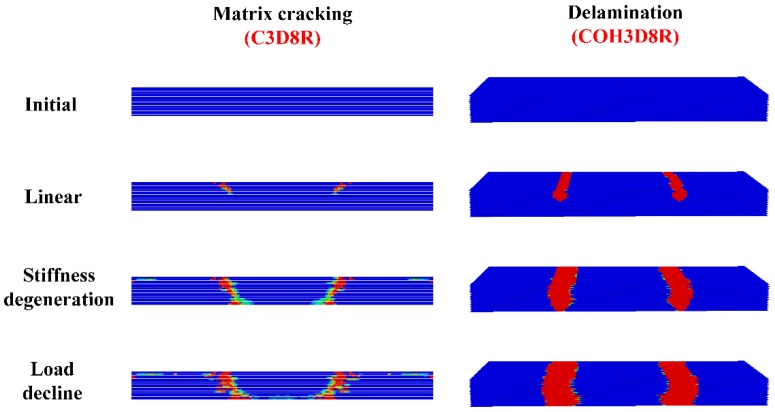
Progressive damage to the lower lap plate of hexagonal bolt joints at 600 °C.

Table 3 shows the critical load of the experiment and simulation, and Figure 14 and Figure 15 show the experimental and simulated load–displacement curves of hexagonal bolt joints and countersunk bolt joints at 600 and 800 °C. For both bolts, the critical load at 800 °C was larger than that at 600 °C. This is consistent with the increase in the strength of C/C composites with temperature within this range. The calculated critical loads of both bolt fasteners at both temperatures were larger than the experimental values. This can be attributed to the degradation of the bearing capacity induced by microscopic defects, such as bubbles and voids, during specimen preparation. These factors, however, were not considered in the finite element simulations. Despite the slight differences, the results of the finite element simulations were highly consistent with the experimental results in terms of the bearing capacity and the failure mode of the specimens. The relation between specimen loading and the load–displacement curves was investigated using the initial load–displacement curve and the cloud chart of the progressive damage in the hexagonal bolt joints at 600 ℃ in the finite element simulations. As shown in Figure 16, the load–displacement curve was divided into A, B, C, and D. The four parts correspond to the four stages shown in Figure 17:

Origin to Point A = initial stage: This concave part was related to the initial gap between the fixture and specimen, depending on the preparation method. On one hand, gaps between the bolt and the opening wall were observed. On the other hand, warp and weft were interlaced in the braiding composites. Due to the heterogeneous thickness of the antioxidant coating on the plate surface, the plate had a large surface roughness and the fixture was not in close contact with the plate. When the load increased, the curve became linear, indicating close contact between the fixture and the plate as well as an effective load transfer. 

Point A to Point B = linear range: The contacts between the fixture and the fastener with the plate were compressed and the fixture and the fastener exerted shear on the plate. No significant deformation of the composite plates and no significant stiffness degradation were observed, except for occasional slight tearing sounds induced by the initial defects. Additionally, the cracks propagated along the thickness at the contact between the lower lap plate and the fixture. Local matrix cracking was observed, as shown in Figure 17. 

Point B to Point C = stiffness degeneration: Frequent and loud tearing sounds were heard, indicating exacerbated damage of the contact between the fixture and the plate. As a result, a slight bending of the plate was observed. B is the inflection point of the curve and corresponds to the yield load of the specimen. When the load increased, plate bending increased and matrix cracking and fiber buckling were observed in areas under tensile sand compressive stress, respectively, which resulted in separation of the matrix and fibers, and delamination. Moreover, cracks emerged along the thickness, the stiffness degraded significantly, and the load was maximal at C. In summary, the BC part refers to the stiffness degeneration part, where the curve was nonlinear. 

Point C to end = load decline: The plate damage accumulated and the delamination cracks propagated along the plate length. Fluctuations were observed because of the large difference between the elastic moduli of the matrix and the fibers. Despite occasional spikes, the load was generally below that at C. This part led to the complete failure of the structure. 

### 3.4. Finite Element Prediction

C/C composites have been widely used in the fabrication of high-temperature components due to their excellent thermal performance. The out-of-plane loading failure and the damage evolution in hexagonal bolt joints and countersunk bolt joints made from C/C composites at 600 and 800 °C were studied. The experimental results were compared with the simulations. 

**Figure 18:**
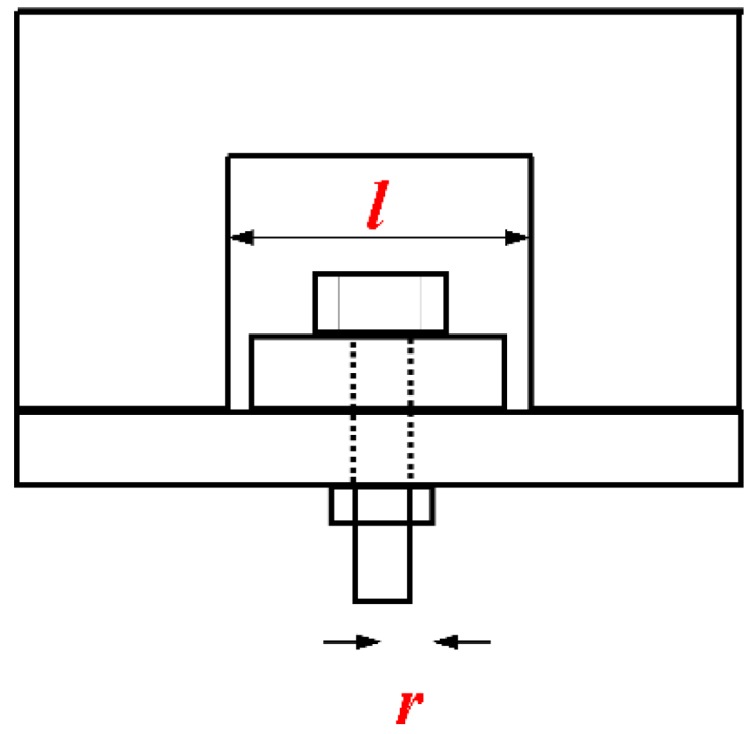
Diagram of variables *r* and *l*.

**Figure 19:**
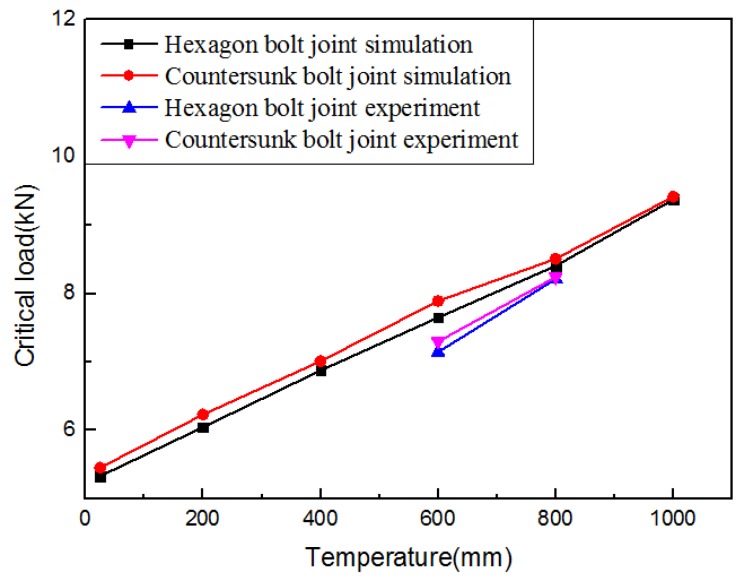
Evolution of the critical load with the temperature.

**Figure 20:**
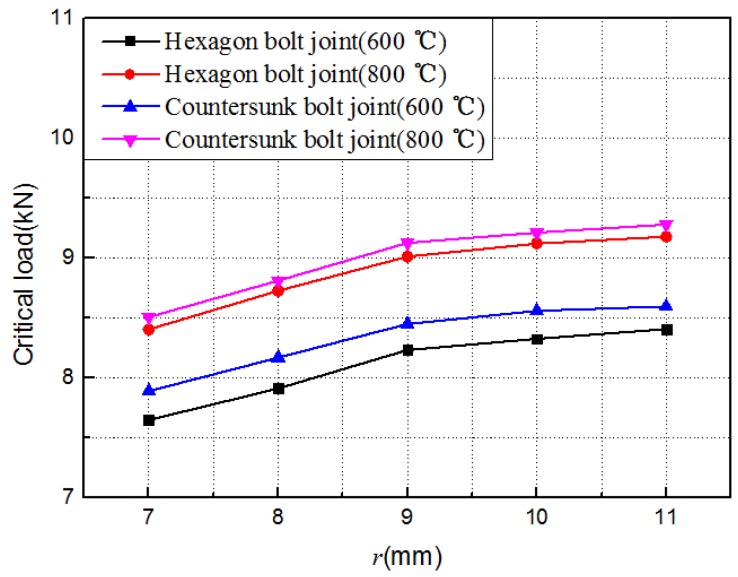
Evolution of the critical load with the radius of the nut *r*.

**Figure 21:**
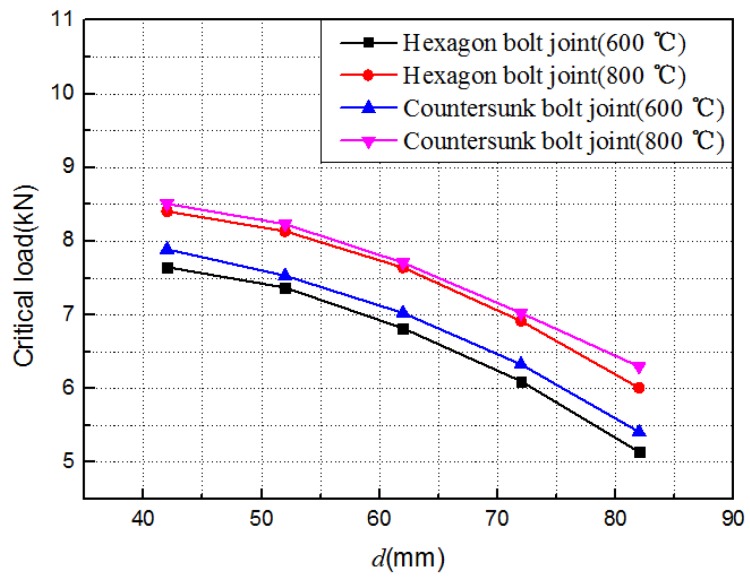
Evolution of the critical load with the distance *d.*

Practically, C/C composite structures of different geometric sizes could work at different temperatures. The influence of the temperature, nut radius *r*, and spacing *d* (in Figure 18) on the out-of-plane load-carrying capacity of C/C composite joints was predicted using the finite element model verified above. The results are shown in Figure 19, Figure 20 and Figure 21.

## 4. Conclusions

In this study, the failure mechanisms and damage evolution of metallic hexagonal head bolt joints and metallic countersunk head bolt joints made from C/C composites experiencing out-of-plane loads at 600 and 800 °C were investigated experimentally and by finite element simulations. The dominant failure modes were determined, the damaged areas were observed using a microscope, and the load–displacement curves and the progressive damage process of the lap plate were studied. The following conclusions can be drawn:

(1) At a specific temperature, the critical loads for the out-of-plane failure of metallic hexagonal bolt joints and metallic countersunk bolt joints made from C/C composites were consistent. The critical loads were higher at 800 °C than at 600 °C, since the strength of the C/C composites increased with the temperature. 

(2) The joints were exposed to a mixed shear/bending failure mode. The damage was mainly distributed on the lower lap plate and mainly originated from cracking and delamination of the matrix. The angle between the damaged area on the side of the lower lap plate and the plate length was approximately 45°. The damage was induced by the contact between the fixture and the nut with the lap plates and propagated towards the opposite side. Additionally, the pulling-out of piercing fibers was observed in areas where dislocations were induced by the shear stress. 

(3) The out-of-plane failure load of the C/C composites bolt joint structure increased with the increase of the nut radius *r*, because the deformation between the two C/C plates was limited as the nut radius *r* increased. However, the out-of-plane failure load of the C/C composites bolt joint structure increased with the increase of the span *d* of the U-shaped clamp. Here, the analysis model has been greatly simplified, and the conclusion is only a preliminary result. Its reliability needs further verification and analysis.

## Figures and Tables

**Table 1:** Performance of the C/C composites at different temperatures.

	RT	600 °C	800 °C		RT	600 °C	800 °C
Density (g/cm^3^)	1.65			Tensile strength $X_{t}$ (MPa)	260	263	271.3
Coefficient of thermal expansion (10–6/°C)	0.19			Compression strength $X_{c}$ (MPa)	176	212	224
Elastic modulus $E_{11}$ (GPa)	85	92.5	95	Tensile strength $Y_{t}$ (MPa)	260	263	271.3
Elastic modulus $E_{22}$ (GPa)	85	92.5	95	Compression strength $Y_{c}$ (MPa)	176	212	224
Elastic modulus $E_{33}$ (GPa)	21	13	10.3	Tensile strength $Z_{t}$ (MPa)	78.6	80.2	84.9
Shear modulus $G_{12}$ (GPa)	20	25.7	27.6	Compression strength $Z_{C}$ (MPa)	326	350	358
Shear modulus $G_{13}=G_{23}$ (GPa)	4	6.1	6.8	Shear strength $S_{12}$ (MPa)	43	51	54.5
Poisson ratio $\nu_{12}$	0.035			Shear strength $S_{13}=S_{23}$ (MPa)	11	16.4	18.2
Poisson ratio $\nu_{13\;}$=$\;\nu_{23}$	0.032						

**Table 2:** Cohesion element parameters.

Temperature	$t_{n}^{0}\;\left(N/mm\right)$	$t_{s}^{0}\;\left(N/mm\right)$	$t_{t}^{0}\;\left(N/mm\right)$	$G^{C}$ (mJ/mm^2^)
RT	20.2	13	13	0.3
600 °C	21.3	13.8	13.8	0.32
800 °C	21.7	14	14	0.33

**Table 3:** The critical loads of the experiment and simulation.

		Hexagon Bolt Joint (N)		Countersunk Bolt Joint (N)	
		600 °C	800 °C	600 °C	800 °C
**Experiment**	1	7155	8028	7294	8186
	2	7134	8396	7302	8296
Simulation		7648	8405	7890	8508
